# NF-κB1 deficiency promotes macrophage-derived adrenal tumors but decreases neurofibromas in HTLV-I LTR-Tax transgenic mice

**DOI:** 10.1371/journal.pone.0303138

**Published:** 2024-05-09

**Authors:** Xinxin Song, Zhaoxia Qu

**Affiliations:** 1 Department of Microbiology and Molecular Genetics, UPMC Hillman Cancer Center, University of Pittsburgh School of Medicine, Pittsburgh, PA, United States of America; 2 Department of Molecular Microbiology and Immunology, Hastings Center for Pulmonary Research, Norris Comprehensive Cancer Center, University of Southern California Keck School of Medicine, Los Angeles, CA, United States of America; Penn State Health Milton S Hershey Medical Center, UNITED STATES

## Abstract

Human T-cell leukemia virus type I (HTLV-I) is an oncogenic virus whose infection can cause diverse diseases, most notably adult T-cell leukemia/lymphoma (ATL or ATLL), an aggressive and fatal malignancy of CD4 T cells. The oncogenic ability of HTLV-I is mostly attributed to the viral transcriptional transactivator Tax. Tax alone is sufficient to induce specific tumors in mice depending on the promotor used to drive Tax expression, thereby being used to understand HTLV-I tumorigenesis and model the tumor types developed in Tax transgenic mice. Tax exerts its oncogenic role predominantly by activating the cellular transcription factor NF-κB. Here, we report that genetic deletion of NF-κB1, the prototypic member of the NF-κB family, promotes adrenal medullary tumors but suppresses neurofibromas in mice with transgenic Tax driven by the HTLV-I Long Terminal Repeat (LTR) promoter. The adrenal tumors are derived from macrophages. Neoplastic macrophages also infiltrate the spleen and lymph nodes, causing splenomegaly and lymphadenopathy in mice. Nevertheless, the findings could be human relevant, because macrophages are important target cells of HTLV-I infection and serve as a virus reservoir *in vivo*. Moreover, the spleen, lymph nodes and adrenal glands are the most common sites of tumor cell infiltration in HTLV-I-infected patients. These data provide new mechanistic insights into the complex interaction between Tax and NF-κB, therefore improving our understanding of HTLV-I oncogenic pathogenesis. They also expand our knowledge and establish a new animal model of macrophage neoplasms and adrenal tumors.

## Introduction

Type I human T-cell leukemia virus (HTLV-I) infection can cause adult T-cell leukemia/lymphoma (ATL or ATLL) and several other diseases [1]. HTLV-I infection can also increase the risk of primary malignant neoplasms other than ATL [2]. HTLV-1 encoded regulatory protein Tax is mainly responsible for the pathogenesis, particularly oncogenesis of HTLV-I. Deletion of Tax from HTLV-I genome leads to loss of its transformation ability, whereas Tax exhibits strong oncogenic ability both *in vitro* and *in vivo* [3,4]. Tax can transform rodent fibroblasts and immortalize human primary T cells *in vitro*, and Tax-transformed lymphoid cells and fibroblasts can form tumors in immunodeficient mice [5–9]. Tax-immortalized T lymphocytes show phenotypes similar to HTLV-I-transformed T cells [4,10]. Moreover, Tax transgenic mice develop tumors, and Tax-mediated T-cell lymphoma in mice closely resembles HTLV-I-induced ATL in human [4,11–18].

Tax exerts its oncogenic role largely through persistently activating nuclear factor-κB (NF-κB), a physiologically indispensable transcription factor whose persistent activation has been linked to nearly all cancer types and inflammation-associated diseases [18–21]. Tax mutants selectively defective in NF-κB activation lose the oncogenic ability [15]. In contrast, Tax mutants that are still able to activate NF-κB retain the transforming capability [15]. Not surprisingly, Tax has been used as a model to study NF-κB and associated pathogenesis, especially, tumorigenesis [4]. NF-κB is a family of transcription factors and consists of five structurally related members in mammalian: NF-κB1, NF-κB2, RelA (also known as p65), RelB and c-Rel [19]. The mechanisms by which Tax deregulates these important transcription factors have been well defined [4,22–26]. Notably, Tax acts as a super scaffold protein, recruiting numerous cellular factors to assemble a large functional complex termed Taxisome that is highly proficient in inducing activation of all five NF-κB members [4,27]. In line with the widely accepted role of NF-κB as the downstream mediator of Tax to drive tumorigenesis, genetic deletion of NF-κB2 significantly prevents the tumorigenesis/neurofibromas in Tax transgenic (*Tax*^+^) mice in which Tax expression is driven by the HTLV-I Long Terminal Repeat (LTR) promoter [28]. Except for NF-κB2, however, the roles of each individual member of the NF-κB family in Tax-mediated tumorigenesis are yet to be dissected. Particularly, genetic evidence is lacking.

Using the same Tax^+^ mice as the model, here we identify an unexpected tumor suppressive function for NF-κB1. Remarkably, while suppressing neurofibromas, genetic deficiency of NF-κB1 promotes immortalization of Tax-expressing macrophages and causes massive infiltration of neoplastic macrophages into the spleen, lymph nodes and adrenal glands, resulting in splenomegaly, lymphadenopathy and adrenomegaly in mice. In addition to advancing our understanding of the complex interaction of NF-κB and Tax in driving tumorigenesis, these studies provide the first evidence revealing that adrenal medullary tumors may be derived from macrophages. They also provide a novel animal model of macrophage neoplasms and adrenal tumors.

## Results

### Induction of adrenal medullary tumors in *Tax*^+^ mice by NF-κB1 deletion

**Fig 1:**
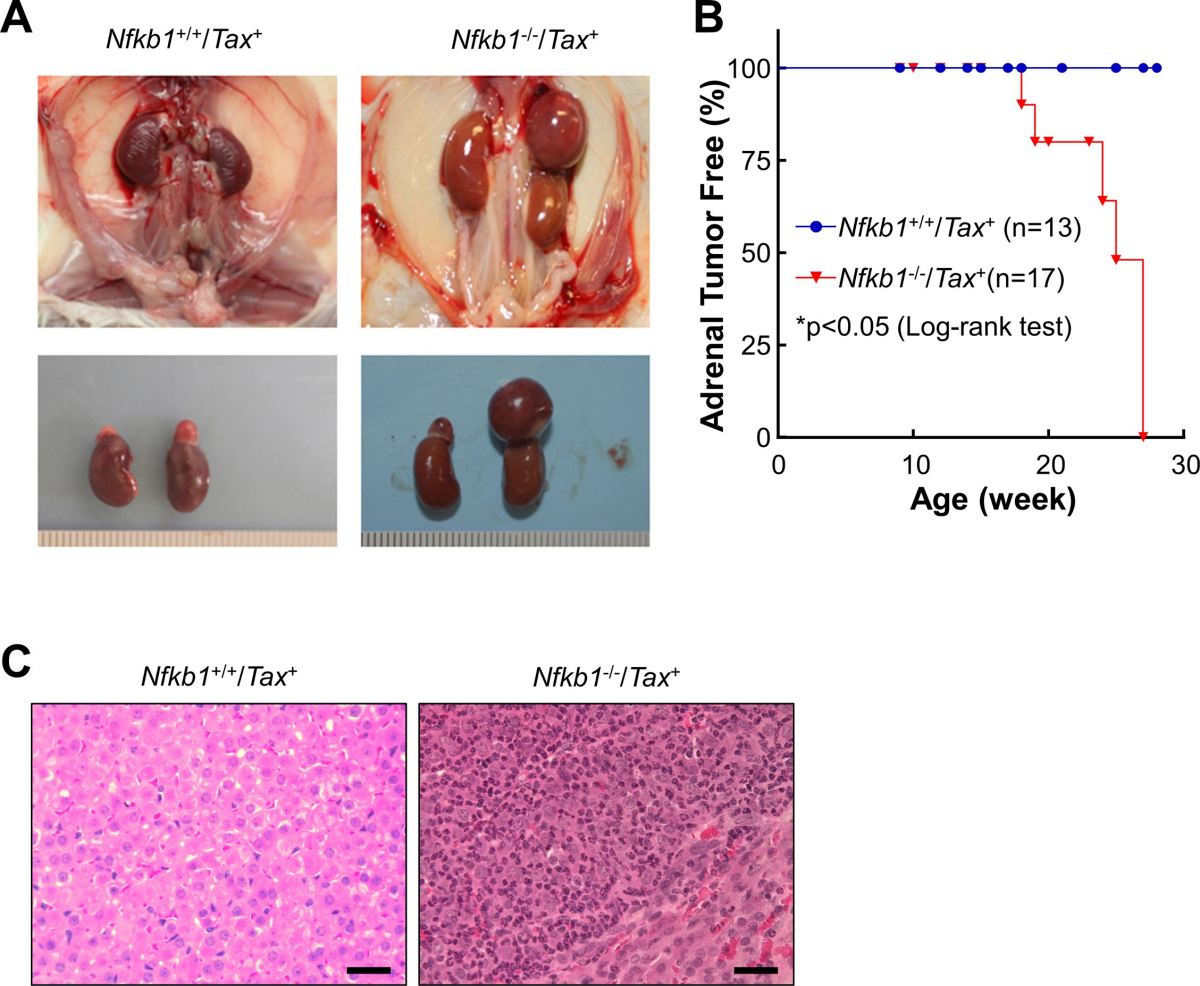
NF-κB1 deletion promotes adrenal medullary tumors in *Tax*^+^ mice. **A**. Representatives of *Tax*^+^ and *Nfkb1*^-/-^/*Tax*^+^ mice at the age of 20 weeks showing induction of adrenomegaly in *Tax*^+^ mice by NF-κB1 deletion. **B.** Adrenomegaly incidence in *Tax*^+^ and *Nfkb1*^-/-^/*Tax*^+^ mice. **C.** H&E staining showing adrenal medullary tumors in *Nfkb1*^-/-^/*Tax*^+^ mice but not *Tax*^+^ mice at the age of 20 weeks. Scale bar: 40 μm.

To determine the role of NF-κB1 in Tax-mediated tumorigenesis, we generated *Nfkb1*^-/-^/*Tax*^+^ mice in which the *Nfkb1* gene is genetically deleted. In line with previous studies showing the role of NF-κB in promoting neurofibromas driven by Tax (28), *Nfkb1* deletion inhibited neurofibroma development in *Tax*^+^ mice (S1 Fig). Surprisingly, *Nfkb1* deletion led to adrenomegaly in about 50% of *Tax*^+^ mice at the age of 25 weeks, whereas no adrenomegaly was observed in Tax^+^ mice with intact *Nfkb1* by the 28-week age (Fig 1A and 1B). Hematoxylin and eosin (H&E) staining revealed a tumor histology in the medulla of the giant adrenal glands of *Nfkb1*^-/-^/*Tax*^+^ mice (Fig 1C). It should be pointed out that *Nfkb1*^-/-^ mice without Tax expression have normal adrenal glands and do not develop any detectable tumors under specific pathogen free conditions [29]. These data suggested that NF-κB1 deletion may promote adrenal medullary tumors in the Tax^+^ mice.

### Macrophage neoplasms in the adrenal glands of *Nfkb1*^-/-^/*Tax*^+^ mice

**Fig 2:**
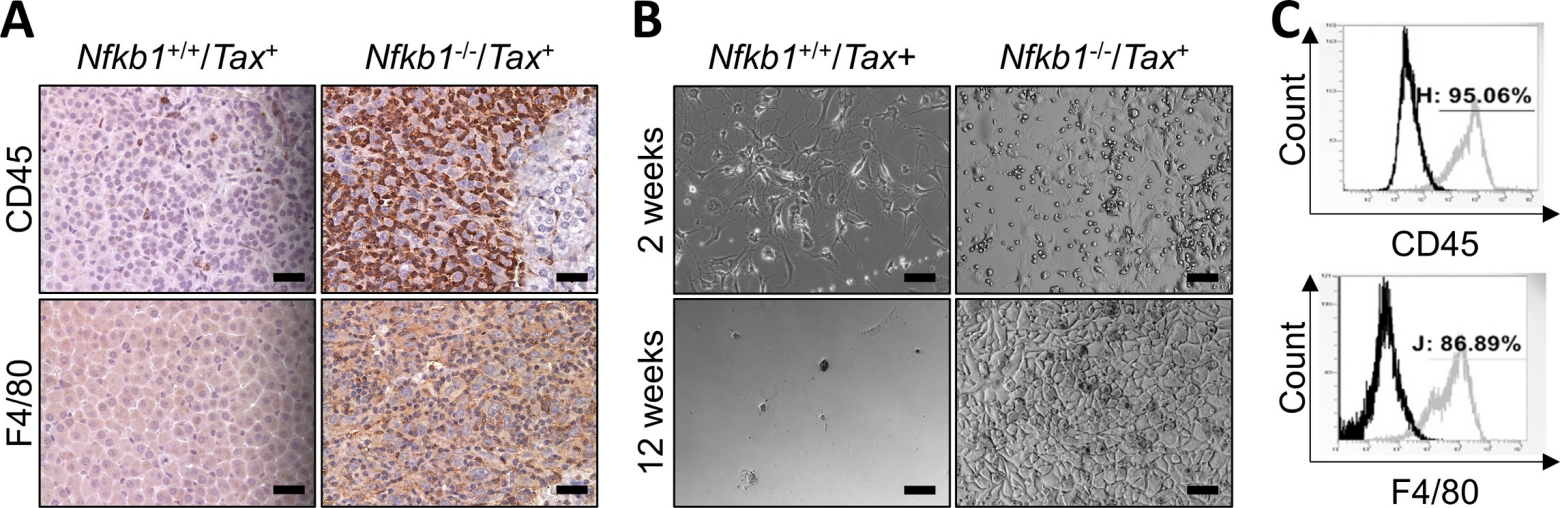
Adrenal medullary tumors in *Nfkb1* ^-/-^/*Tax*^+^ mice are macrophage-derived. **A.** IHC staining showing CD45^+^ and F4/80^+^ adrenal medullary tumors in *Nfkb1*^-/-^/*Tax*^+^ mice at the age of 20 weeks. Scale bar: 40 μm. **B.** *In vitro* culture showing the immortalization of primary adrenal gland cells of *Nfkb1*^-/-^/*Tax*^+^ mice but not *Tax*^+^ mice at the age of 20 weeks. Representative morphology of primary adrenal gland cells at 2 weeks and 12 weeks post *in vitro* culture were shown. Scale bar: 40 μm. **C.** Flow cytometric analysis showing CD45^+^ and F4/80^+^ primary adrenal gland cells of *Nfkb1*^-/-^/*Tax*^+^ mice 12 weeks post *in vitro* culture.

Our immunohistochemistry (IHC) staining further revealed that the tumor cells in the adrenal glands of *Nfkb1*^-/-^/*Tax*^+^ mice were positive for the immune cell marker CD45 and the macrophage marker F4/80 (Fig 2A), suggesting that macrophages were the cells of origin of the adrenal medullary tumors in the *Nfkb1*^-/-^/*Tax*^+^ mice. To validate the data, we cultured *in vitro* the primary adrenal cells of *Tax*^+^ and *Nfkb1*^-/-^/*Tax*^+^ mice under the same normal culture conditions. Adrenal cells from *Tax*^+^ mice started to die after 2 weeks of culture, and all cells died by 12 weeks (Fig 2B, left panels). In stark contrast, adrenal cells from *Nfkb1*^-/-^/*Tax*^+^ mice kept growing after 12 weeks (Fig 2B, right panels), indicating that those cells were immortalized. Consistent with the IHC staining of the adrenal glands of *Nfkb1*^-/-^/*Tax*^+^ mice, our flow cytometry analysis showed that the immortalized cells were macrophages (Fig 2C). These data suggested that NF-κB1 deletion induces macrophage neoplasms in the adrenal glands of the *Tax*^+^ mice.

### Association of Tax expression with adrenal medullary tumors in *Nfkb1*^-/-^/*Tax*^+^ mice

**Fig 3:**
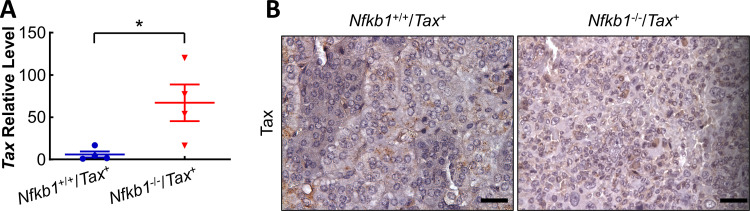
Adrenal medullary tumors in *Nfkb1*^-/-^/*Tax*^+^ mice are associated with elevated Tax expression. **A.** RT-qPCR showing a significantly higher level of *Tax* mRNA in the adrenal glands of *Nfkb1*^-/-^/*Tax*^+^ mice. *Nfkb1*^-/-^/*Tax*^+^ and *Tax*^+^ mice used were at the age of 16–25 weeks. *Tax* mRNA levels in each sample were normalized to the mRNA level of *Actin*. **B.** IHC staining showing more Tax protein expression in the adrenal glands of *Nfkb1*^-/-^/*Tax*^+^ mice compared to those of *Tax*^+^ mice at the same age of 20 weeks. Scale bar: 40 μm.

Given the powerful transforming ability of Tax [1,4], we compared both the RNA and protein levels of this viral oncoprotein in the adrenal glands of *Tax*^+^ and *Nfkb1*^-/-^/*Tax*^+^ mice. Our quantitative real-time reverse transcription–polymerase chain reaction (RT-qPCR) showed a significantly higher level of the Tax mRNA in the adrenal glands of *Nfkb1*^-/-^/*Tax*^+^ mice (Fig 3A). Consistently, more Tax protein expression was detected in the adrenal glands of *Nfkb1*^-/-^/*Tax*^+^ mice by IHC staining assays (Fig 3B). These data suggested that NF-κB1 deletion induces macrophage-derived adrenal tumors in the *Tax*^+^ mice by inducing and/or maintaining expression of the Tax oncoprotein.

### Malignant lymphadenopathy and splenomegaly in *Nfkb1*^-/-^/*Tax*^+^ mice

**Fig 4:**
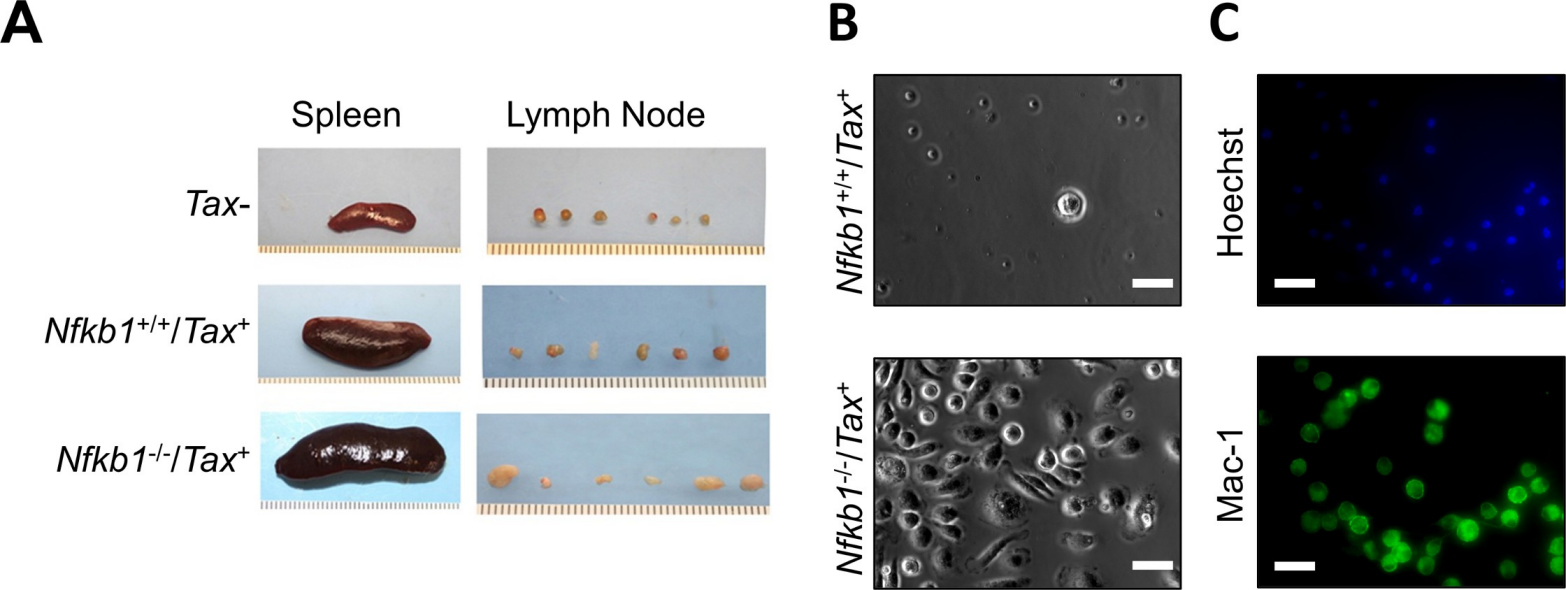
*Nfkb1* ^-/-^/*Tax*^+^ mice have malignant lymphadenopathy and splenomegaly. **A.** Representative spleens and lymph nodes of WT, *Tax*^+^ and *Nfkb1*^-/-^/*Tax*^+^ mice at the age of 20 weeks. **B.** *In vitro* culture showing the immortalization of primary lymph node cells from *Nfkb1*^-/-^/*Tax*^+^ mice but not *Tax*^+^ mice at the age of 20 weeks. Representative morphology of primary lymph node cells at 4 weeks post *in vitro* culture were shown. Scale bar: 40 μm. **C.** IF assays showing Mac-1^+^ primary lymph node cells of *Nfkb1*^-/-^/*Tax*^+^ mice 4 weeks post *in vitro* culture. Scale bar: 40 μm.

In line with previous studies [11,28], enlarged spleens and lymph nodes were observed in the *Tax*^+^ mice compared to those matched wild type (WT) mice (Fig 4A). Remarkably, the spleen and lymph nodes of *Nfkb1*^-/-^/*Tax*^+^ mice were even much bigger. More importantly, almost all cells from the lymph nodes of the *Tax*^+^ mice died within 4 weeks of culture *in vitro*, whereas many lymph node cells from the *Nfkb1*^-/-^/*Tax*^+^ mice survived and kept growing (Fig 4B). Immunofluorescent (IF) staining showed the survived cells were positive for macrophage-1 antigen (Mac-1, also known as macrophage integrin or integrin αMβ2), a macrophage marker (Fig 4C). These data suggested that NF-κB1 deletion not only promotes macrophage transformation but also cause massive infiltration of neoplastic macrophages into the lymph nodes and spleen of the *Tax*^+^ mice.

## Discussion

Macrophages are important target cells and act as a virus reservoir of HTLV-I infection *in vivo* [30–33]. However, it remains unknown whether HTLV-I infection causes neoplastic transformation of macrophages, as it does for T-cell tumorigenesis. Similarly, whether Tax can immortalize macrophages is also unknown. The studies above clearly demonstrated, for the first time, that Tax is capable to transform macrophages into cancerous cells at least in mice in which NF-κB1 is genetically deleted. These studies also identify a previously unidentified tumor suppressive function of NF-κB in HTLV-I/Tax-mediated tumorigenesis.

Macrophage neoplasm in *Nfkb1*^-/-^/*Tax*^+^ mice is a systemic malignancy that seems originally derived from adrenal glands, as adrenomegaly occurs earlier than lymphadenopathy and splenomegaly in the mice. It is noteworthy that adrenal glands have been identified as the primary tumor site in HTLV-I-infected patients, although it was proposed that tumor cells were derived from T cells in the adrenal glands [34]. However, the report failed to show that tumor cells expressed the T-cell marker CD3, CD4 or CD8, although tumor cells were positive for CD45 but not the B-cell marker CD20. It is thus of interest to examine whether the human primary adrenal tumors were derived from macrophages like those in *Nfkb1*^-/-^/*Tax*^+^ mice. Of note, adrenal glands, lymph nodes and spleens are the most common infiltration sites of ATL cells [34,35]. While this would be true in most HTLV-I-infected patients, an extreme caution should be taken to ensure whether the infiltrated tumor cells are derived from T cells or macrophages in each clinical case.

One important function of NF-κB1 in suppressing Tax-mediated neoplastic transformation of macrophages is to selectively prevent Tax expression in macrophages, particularly those resided in adrenal glands, as *Nfkb1* deletion has no obvious effect on Tax expression in the spleen and lymph nodes of *Tax*^+^ mice (S2 Fig). NF-κB1 is known to repress gene transcription, directly by the homodimer of its mature form p50 and/or indirectly by its precursor form p105 as the inhibitor of NF-κB [4]. Another potential mechanism may involve the NF-κB-independent function of p105 in stabilizing the kinase tumor progression locus 2 (Tpl2, also known as COT or MAP3K8) [29,36]. In this regard, the p105/Tpl2 axis has been demonstrated to be critical in lung cancer suppression [29]. Genetic deletion of NF-κB1 or Tpl2 promotes lung tumorigenesis in mouse models, whereas reconstitution of p105 or Tpl2 inhibits the tumorigenicity of NF-κB1 deficient lung tumor cells [29]. Nevertheless, the studies here provide another new layer of Tax inhibition by the host for the prevention of HTLV-I pathogenesis. In contrast to the well-defined mechanisms by which Tax hijacks cellular factors for pathogenesis, how most HTLV-I infected people sovereign Tax to keep asymptomatic is poorly understood. Especially, macrophage neoplasms have rarely been reported in peoples infected with HTLV-I. PDZ-LIM domain-containing protein 2 (PDLIM2), an essential tumor suppressor and immunomodulator, was the first cellular protein identified to negatively regulate Tax [20,37–46]. It directly binds to and promotes Tax ubiquitination and proteasomal degradation, thereby blocking HTLV-I/Tax-mediated tumorigenesis [37]. NF-κB1 is the second identified cellular factor that negatively regulates Tax. Interestingly, NF-κB1 suppresses Tax at the RNA level. So, the host uses complementary mechanisms to control this viral oncoprotein at both RNA and protein level.

In line with previous studies [11,12,28], *Tax*^+^ mice develop neurofibromas in the ear, nose and tail. However, the genetic deletion of NF-κB1 fails to promote neurofibromas in *Tax*^+^ mice. Instead, *Nfkb1* deletion significantly delays neurofibroma formation in the mice, indicating a promoting role of NF-κB1 in Tax-driven neurofibromatosis. Of note, genetic deletion of NF-κB*2* also delays the development of neurofibroma in *Tax*^+^ mice, though at a greater extent [28]. It should also be pointed out that no macrophage neoplasms are detected in *Nfkb2*^-/-^/*Tax*^+^ mice [28]. These genetic studies demonstrate the mutually independent contribution of NF-κB1 and NF-κB2 in Tax-mediated neurofibromas. They also reveal a unique role for NF-κB1 in suppressing Tax expression and subsequent macrophage transformation and adrenal tumor formation.

In summary, the present studies demonstrate that NF-κB1 promotes neurofibromas but prevents the neoplastic transformation of macrophages and the formation of adrenal medullary tumors induced by the HTLV-I oncoprotein Tax. They provide new mechanistic insights into the complex interactions of Tax with NF-κB and the host, therefore enlightening our understanding of HTLV-I pathogenesis. They also improve our knowledge and offer a new animal model of macrophage neoplasms and adrenal tumors.

## Materials and methods

### Animals

*Nfkb1*^-/-^ and *Tax*^+^ mice have been described before [28,29]. *Nfkb1*^-/-^ mice were originally purchased from Jackson Laboratory (Bar Harbor, ME, USA). *Tax*^+^ mice were generous gifts from J.E. Green [11]. *Nfkb1*^-/-^ and *Tax*^+^ mice were bred to generate *Nfkb1*^-/-^/*Tax*^+^ mice. All animals were maintained under specific pathogen-free conditions and used according to protocols approved by the IACUC of the University of Pittsburgh and the University of Southern California. All research staff have completed laboratory animal care and use training before working with mice. Mouse health and behavior were monitored at least once per day. The mice with pain and distress were treated according to attending veterinarian’s suggestions. Mice were euthanized at the indicated time points or when they exhibit severe difficulty in breathing or moving, significant weight loss (exceeding 20% of the body weight), moribund states of arousal, ulcerated or necrotic tumor, a visible tumor of size exceeding 1.5 cm in any dimension, or the combined volume of visible tumors exceeds 2000 mm^3^ (2.0 cm^3^), or body condition score (BCS) reaches <2/5. Euthanasia was performed by CO_2_ inhalation followed by cervical dislocation.

### Cell culture

Primary cells of adrenal glands and lymph nodes were cultured in Dulbecco modified Eagle medium (DMEM) supplemented with 10% fetal bovine serum (FBS) and 2 mM l-glutamine for the indicated time period. The culture mediums were changed every two days.

### Histology and IHC assays

Mouse adrenal glands were excised, fixed in formalin, embedded in paraffin, and cut into 4-μm-thick sections. Sections were stained with H&E or subjected to sequential incubations with the indicated primary antibodies, biotinylated secondary antibodies and streptavidin-HRP as described before [47–49]. Antibodies used are listed in S1 Table.

### IF analysis

Cells were fixed, permeabilized, and subsequently incubated with Mac-1 antibody, followed by FITC-conjugated secondary antibody. Cells were also counterstained with Hoechst for nuclear staining. Stained proteins and their subcellular localizations were detected using a fluorescence microscope [50].

### RT-qPCR analysis

Mouse adrenal glands were subjected to RNA extraction, RNA reverse transcription and qPCR as described [51–53]. Primers for qPCR are listed in S2 Table.

### Flow cytometry analysis

The indicated cells were fixed with paraformaldehyde (2%) and permeablized with saponin (0.5%), or directly treated with the indicated antibodies. Data were acquired using FACSCalibur (BD Biosciences) and analyzed using CellQuest software (Becton Dickinson) as described [54–57]. Antibodies used are listed in S1 Table.

### Statistics

Student’s *t* test (2 tailed, unpaired) was used to assess significance of differences between 2 groups. All bars in figures represent mean ± SEM. *P* values less than 0.05 and 0.01 were considered statistically significant and highly statistically significant, respectively).

## Supporting information

S1 Fig NF-κB1 deletion delayed neurofibroma development in Tax+ mice. (PDF)

S2 Fig NF-κB1 deletion has no effect on Tax expression in the spleen and lymph nodes of Tax+ mice. (PDF)

S1 Table Antibodies used. (PDF)

S2 Table Primers used. (PDF)

S1 File (XLSX)
