# Supplementary material for: NF-κB1 deficiency promotes macrophage-derived adrenal tumors but decreases neurofibromas in HTLV-I LTR-Tax transgenic mice
Source: PLoS One. 2024 May 9;19(5):e0303138. doi: 10.1371/journal.pone.0303138 (PMC11081228; doi:10.1371/journal.pone.0303138)
Supplement: S1 Fig — (PDF) [file pone.0303138.s001.pdf]

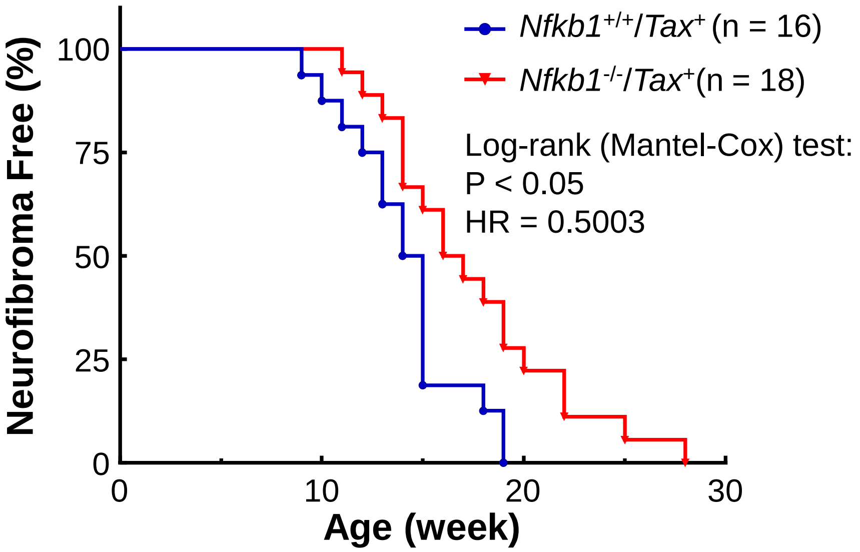

Fig S1. NF-κB1 deletion delayed neurofibroma development in *Tax*⁺ mice.
Neurofibroma incidence in *Nfkb1*$^{+/+}$/*Tax*$^{+}$ and *Nfkb1*$^{-/-}$/*Tax*$^{+}$ mice.
