# Supplementary material for: NF-κB1 deficiency promotes macrophage-derived adrenal tumors but decreases neurofibromas in HTLV-I LTR-Tax transgenic mice
Source: PLoS One. 2024 May 9;19(5):e0303138. doi: 10.1371/journal.pone.0303138 (PMC11081228; doi:10.1371/journal.pone.0303138)
Supplement: S2 Fig — (PDF) [file pone.0303138.s002.pdf]

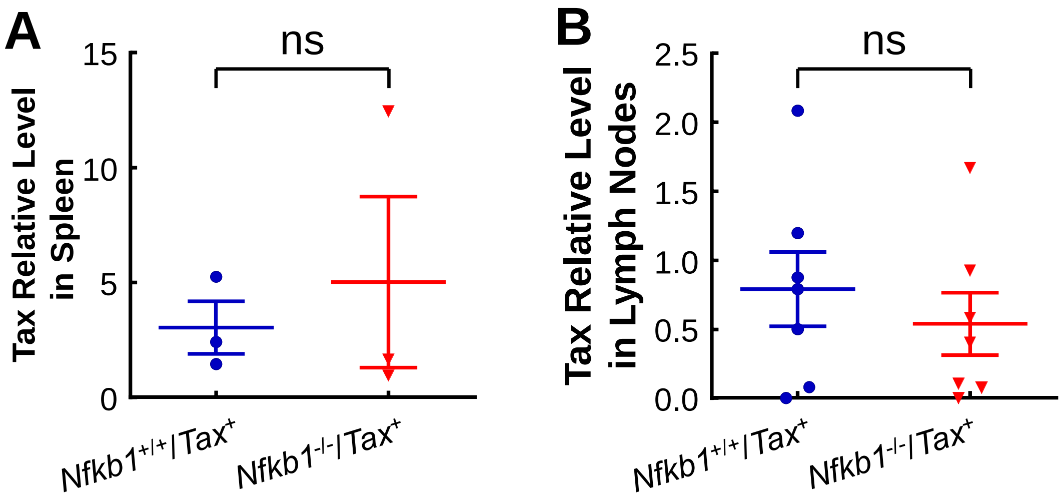


**Fig S2. NF-κB1 deletion has no effect on Tax expression in the spleen and lymph nodes of *Tax*⁺ mice.**

RT-qPCR showing a similarly low level of *Tax* mRNA in the spleen and lymph nodes of *Nfkb1*$^{+/+}$/*Tax*$^{+}$ and *Nfkb1*$^{-/-}$/*Tax*$^{+}$ mice. *Nfkb1*$^{+/+}$/*Tax*$^{+}$ and *Nfkb1*$^{-/-}$/*Tax*$^{+}$ mice used were at the age of 16-25 weeks. *Tax* mRNA levels in each sample were normalized to the mRNA level of A*ctin*.
