# Supplementary material for: NF-κB1 deficiency promotes macrophage-derived adrenal tumors but decreases neurofibromas in HTLV-I LTR-Tax transgenic mice
Source: PLoS One. 2024 May 9;19(5):e0303138. doi: 10.1371/journal.pone.0303138 (PMC11081228; doi:10.1371/journal.pone.0303138)
Supplement: S1 Table — (PDF) [file pone.0303138.s003.pdf]

Supplemental Table S1. Antibodies Used

Antibody	Clone	Catalog number	Company	Usage	Purpose
Anti-CD45	30-F11, Rat IgG2b, κ	550539	BD Biosciences, USA	1:25	IHC
Anti-F4/80	Cl:A3-1, Rat IgG2b	MCA497G	Bio-Rad, Hercules, CA, USA	1:200	IHC
Anti-Tax	168B17-46-49		AIDS Research and Reference Program (NIAID)	1:20	IHC
Anti-CD45 PE	30-F11, Rat IgG2b, κ	553081	BD Biosciences, USA	1.0 µl per sample	FACS
Anti-F4/80 APC	BM8, Rat IgG2a, κ	17-4801	Thermo Fisher Scientific, Waltham,MA, USA	1.0 µl per sample	FACS
Anti-CD11b (Mac-1)	M1/70, Rat IgG2b, κ	14-0112	Thermo Fisher Scientific, Waltham,MA, USA	1:200	IF
Anti-CD16/CD32	93, Rat IgG2a, λ	14-0161	Thermo Fisher Scientific, Waltham,MA, USA	1.0 µl per sample	FACS
Rat IgG2b κ Isotype Control, PE	eB149/10H5,	12-4031	Thermo Fisher Scientific, Waltham,MA, USA	0.625 µl per sample	FACS
Rat IgG2a κ Isotype Control, APC	eBR2a	17-4321	Thermo Fisher Scientific, Waltham,MA, USA	0.625 µl per sample	FACS
Goat anti-rat IgG-FITC		sc-2011	Santa Cruz Biotechnology, Dallas, TX,USA	1:200	IF
