# Supplementary material for: NF-κB1 deficiency promotes macrophage-derived adrenal tumors but decreases neurofibromas in HTLV-I LTR-Tax transgenic mice
Source: PLoS One. 2024 May 9;19(5):e0303138. doi: 10.1371/journal.pone.0303138 (PMC11081228; doi:10.1371/journal.pone.0303138)
Supplement: S2 Table — (PDF) [file pone.0303138.s004.pdf]

Supplemental Table S2. Primers Used

Gene	Species	Accession number	Forward (5' to 3')	Reverse (5' to 3')	Usage
Actin	Mouse	NM_007393.3	ACCCGCGAGCACAGCTTCTTTG	CTTTGCACATGCCGGAGCCGTTG	RT-PCR
Tax	HTLV-1	NC_001436.1	CTGTCCAGAGCATCAGATCACCTGG	GGTTCCATGTATCCATTTCGGAAGG	RT-PCR
